# Biomarker Discovery in Atherosclerotic Diseases Using Quantitative Nuclear Magnetic Resonance Metabolomics

**DOI:** 10.3389/fcvm.2021.681444

**Published:** 2021-07-28

**Authors:** Shuai Ma, Mingfeng Xia, Xin Gao

**Affiliations:** ^1^Department of Endocrinology and Metabolism, Zhongshan Hospital, Fudan University, Shanghai, China; ^2^Fudan Institute for Metabolic Diseases, Shanghai, China; ^3^Human Phenome Institute, Fudan University, Shanghai, China

**Keywords:** quantitative nuclear magnetic resonance, metabolomics, biomarker, lipoprotein, atherosclerosis, cardiovascular disease

## Abstract

Despite great progress in the management of atherosclerosis (AS), its subsequent cardiovascular disease (CVD) remains the leading cause of morbidity and mortality. This is probably due to insufficient risk detection using routine lipid testing; thus, there is a need for more effective approaches relying on new biomarkers. Quantitative nuclear magnetic resonance (qNMR) metabolomics is able to phenotype holistic metabolic changes, with a unique advantage in regard to quantifying lipid-protein complexes. The rapidly increasing literature has indicated that qNMR-based lipoprotein particle number, particle size, lipid components, and some molecular metabolites can provide deeper insight into atherogenic diseases and could serve as novel promising determinants. Therefore, this article aims to offer an updated review of the qNMR biomarkers of AS and CVD found in epidemiological studies, with a special emphasis on lipoprotein-related parameters. As more researches are performed, we can envision more qNMR metabolite biomarkers being successfully translated into daily clinical practice to enhance the prevention, detection and intervention of atherosclerotic diseases.

## Introduction

Cardiovascular disease (CVD) remains the leading cause of morbidity and mortality around the world (1). Of the 55.95 million global deaths in 2017, 17.79 million (31.8%) were due to CVD (2). Atherosclerosis (AS), as the underlying pathogenesis, starts early in life and takes decades to develop into serious vascular occlusion, thus, effective detection and well-understanding of the diseases would be valuable for primary prevention (3).

Although definite mechanisms of atherogenic process continue to be intensively investigated, lipids or lipoproteins are known to play pivotal roles (4). Circulating low-density lipoprotein (LDL) and other ApoB-containing lipoproteins constitute a key driver of plaque formation through their interaction with monocyte-derived macrophages in the arterial intima. Due to the accumulation of retained lipoproteins, macrophage cells and extracellular matrix, the vascular lesions gradually enlarge and progress into more complex arterial plaques (5). Since dyslipidemia is a recognized risk factor for atherosclerosis, routine evaluation of lipoprotein level offers a valid contribution to stratify atherosclerotic risk (6). However, the standard lipid panel is not always sensitive and specific enough for the identification of patients at risk.

Low-density lipoprotein cholesterol (LDL-C) is a well-established risk factor and the primary intervention target (7–9). However, individuals who develop atherosclerotic diseases may have “optimal” LDL-C levels (10, 11), especially subjects with insulin resistance (12, 13). Additionally, patients who receive cholesterol-lowering treatment and reach satisfactory LDL-C targets still develop CVD (14). This is partly due to other nonlipid risk factors, such as hypertension, cigarette smoking, inactivity, obesity, and type 2 diabetes (T2DM). However, it is likely that other lipid or lipoprotein factors also play a part in the atherogenic process.

Many large epidemiological studies have supported that triglycerides (TGs) correlate with CVD risk (15–17), although the association is sometimes attenuated with adjustment for other traditional risk factors (18). However, increasing evidence now has highlighted the importance of triglyceride-rich lipoproteins (TRLs) and their remnants, both in fasting and nonfasting periods (19–22). Populations with mild-to-moderately elevated nonfasting TG levels have increased risks for ischemic stroke, myocardial infarction, ischemic heart disease, and all-cause mortality (23–25). Mendelian randomization studies also demonstrate that TRLs are causally associated with CVD and all-cause mortality (26). Thus, new insights strongly suggest that TRLs are independent CVD factors; however, the discrepancies in atherogenicity among their subclasses and lipid content are still an area of continuing investigation.

Additionally, there exists a widely held view that high-density lipoprotein cholesterol (HDL-C) levels causally relate to AS. Nevertheless, the EPOCH-JAPAN (Evidence for Cardiovascular Prevention from Observational Cohorts in Japan) study (27) and CANHEART (Cardiovascular Health in Ambulatory Care Research Team) study (28) both presented a U-shaped relationship between HDL-C and CVD mortality. In addition, elevated HDL-C from cholesterol ester transfer protein (CETP) inhibitors failed to lower CVD risk (29, 30), and gene variants altering HDL-C levels in Mendelian randomization studies were not necessarily associated with CVD (31). Instead, it has been proposed that indexes of HDL structure and function may better mediate the atheroprotective effects of HDL (32).

Hence, the standard metrics in routine lipid testing are only part of lipoprotein factors, which cannot account for all CVD risks. Lipoprotein is known to be a lipid-protein conglomerate possessing complex structure, composition and function (33). Chylomicron containing ApoB-48 is secreted from the intestine, whereas very low density lipoproteins (VLDL) containing ApoB-100 is synthesized in hepatocytes and then enters the systemic circulation. TGs in VLDL are hydrolyzed by lipoprotein lipase (LPL), resulting in small, cholesterol-enriched VLDL remnants and IDL (34). IDL particles can be further converted into LDL by hepatic lipase (HL), and both of them can be removed by the interaction of apolipoproteins with hepatic LDL receptor (LDLR) (35). The HDL metabolism begins with secretion of small discoidal protein-phospholipid complex mainly from liver. The nascent HDL removes cholesterol from peripheral tissues, and increasingly matures into a larger spherical HDL particle. In summary, the metabolic processes of lipoproteins are very complex, which involve dynamic changes of cholesterol, triglyceride, phospholipid and apolipoprotein in various lipoprotein subclasses (36). More detailed lipoprotein profiles based on advanced detection methods are necessary to identify new biomarkers of AS and its clinical manifestations (37–39).

Metabolomics is able to measure the global metabolic status in cells, tissues or biological fluids (40). Being downstream of biometabolic processes, the metabolome can filter out nonfunctional effects and greatly amplify small functional changes at the genetic or protein expression levels (41). Quantitative nuclear magnetic resonance (qNMR) metabolomics enables rapid, nondestructive, reproducible and high-throughput quantifications of lipoproteins, lipids and molecular metabolites with uncomplicated sample preparations (42). Since mass spectrometry (MS) cannot analyze protein-lipid complexes, qNMR metabolomics occupies a very important position in lipoprotein testing. It has also been demonstrated that qNMR lipoprotein detection is unaffected by frozen storage and multiple freeze-thaw cycles (43). Because of these advantages, qNMR metabolomics paves the way for large population-based studies and, therefore, provides a distinct perspective on atherosclerotic diseases (44, 45).

Over the past two decades, accumulating evidence has suggested that qNMR metabolomics can serve as a promising strategy to discover new biomarkers in atherosclerotic diseases (46, 47). The qNMR-based lipoproteins with differing sizes and densities have diverse vascular effects (48, 49). The profiling of metabolic status holds promise of unraveling the pathological mechanisms underlying atherosclerosis. In addition, qNMR metabolomics may better reflect atherogenesis than traditional lipid testing (48, 50, 51). The quantification of circulating metabolites identifies changes prior to the onset of overt disease, and hereby contributes to earlier and more accurate cardiovascular risk assessment.

Despite such a large number of studies in this field, no literature has systematically reviewed the qNMR-based metabolite biomarkers in detail. In this review, we intend to (1) describe the detection and analytical principle of qNMR metabolomics; (2) summarize the epidemiological findings that seek novel biomarkers for AS and CVD using qNMR metabolomics, focusing on lipoprotein and lipid; (3) discuss the significance of identifying qNMR biomarkers; (4) illustrate the current challenges and future directions in this field.

## Quantitative NMR-Based Metabolomics

In 1983, Nicholson et al. (52, 53) pioneered the application of NMR spectroscopy to study multicomponent metabolic composition. NMR-based metabolomics identifies metabolites subjected to a magnetic field by characteristic chemical shifts in resonance frequency. Quantitative NMR metabolomics, also known as metabolite profiling, offers transparent biological information about metabolite identification and absolute quantitation (54). The most versatile and widely used method is ^1^H qNMR. Each molecule with hydrogen atoms gives a characteristic signal with a shape that is quantum mechanically distinctive, and the area is proportional to the concentration of the molecule (55, 56). Using standard equipment, qNMR can resolve the concentrations of target molecules down to the micromolar range. By enhancing or attenuating different signals with multiple pulse programs, qNMR is able to optimize large or small molecules (57). Individual molecular signals may overlap, but current linefitting methods relying on molecule-specific model lineshapes and regression modeling can robustly handle part of the overlapping information (58, 59).

It is also widely known that qNMR is suitable for detecting and quantifying lipoproteins because the chemical shifts fundamentally reflect the physical structure of the particle (60, 61). Lipoproteins give rise to a series of broad peaks in spectral regions that are superimposed on the very broad envelope from proteins such as albumin and the sharp peaks of small-molecule metabolites (62). Among them, the shapes of two peaks, i.e., the terminal CH3 group peak and the long-chain (CH2)n peak of the fatty acyl groups, can reflect the differences derived from lipid chemical heterogeneity and lipoprotein particle size (63). According to the size of the particle, the lipid methyl and methylene moieties in lipoproteins resonate at different frequencies, with smaller particles resonating at lower frequencies. Therefore, the concentrations of lipoproteins can be quantitated when decomposing the methyl and methylene signals of the core lipids into individual signals or using statistical methods to estimate the entire envelope (64).

To further determine lipoprotein parameters, research teams have developed various fitting models to cover different lipoprotein subfractions categorized by size or density. The research team led by Otvos first developed lipoprotein quantification in 1991 (65); they detected the particle numbers and sizes of large, medium, and small subclasses of VLDL, LDL, and HDL separately. The group then created a simplified LipoProfile® panel, which has been commercially available from LipoScience Inc. (Raleigh, North Carolina) since 1997 (66). The Finnish research team led by Ala-Korpela determined the particle numbers and sizes of 14 lipoproteins (CM, 5 VLDL, 1 IDL, 3 LDL, and 4 HDL) (67). A Spanish research team developed a Liposcale panel, which was reported to be more accurate than the LipoProfile (68). The spectrometer manufacturer company Bruker BioSpin also offers lipoprotein analysis. The latest method is capable of quantifying over 100 parameters, including 16 lipoprotein subclasses (5 VLDL, 1 IDL, 6 LDL, and 4 HDL) and their compositional components (total cholesterol, free cholesterol, triglyceride, phospholipid, apolipoprotein etc.) (62). It also reports 41 low-molecular-weight metabolites in units of mole, mildly extending the metabolic profiles.

## Biomarkers for Atherosclerotic Diseases Using qNMR Metabolomics

Herein, drawing on discoveries in population-based studies, we describe the qNMR biomarkers from a perspective of three main categories of lipoproteins (i.e., LDL, VLDL, and HDL), along with some molecular metabolites. We provide a tabular overview of qNMR-based potential lipoprotein biomarkers (Table 1). We also summarize the epidemiological studies of biomarker discovery in subclinical atherosclerosis (Supplementary Table 1) and cardiovascular disease (Supplementary Table 2) using qNMR metabolomics in recent 5 years.

**Table 1:** Lipoprotein biomarkers of atherosclerotic diseases identified in epidemiological studies using quantitative NMR metabolomics.

**Biomarker**	**Full name**	**Unit**	**Potential ability**	**Hypothesized mechanisms**	**Need for further study**
**Low-density lipoprotein (LDL)**					
LDL-P	LDL particle number	nmol/L	LDL-P is a “reasonable measure” for atherosclerotic diseases. When LDL-C and LDL-P are discordant, LDL-P indicates additional risk information. The LipoProfile panel including LDL-P has entered clinical practice in United States.	Increased LDL-P, within the same level of LDL-C, reflects elevated small, dense LDL particles which is more atherogenic.	Although LDL-P in LipoProfile has achieved initial clinical transformation, the clinical utilization rates and practical benefits remain unclear so far.
Small LDL-P	Small LDL particle number	nmol/L	Small and dense LDL (sdLDL) is a well-recognized atherogenic indicator, which has been highlighted by many guidelines.	Smaller LDL has a lower affinity for receptor, greater affinity for arterial wall, and is more prone to oxidation.	The precipitating factors for elevated small LDL-P under various disease environments need to be explored.
Large LDL-P	Large LDL particle number	nmol/L	Some studies regarded large LDL as important as sdLDL. Failure to adjust the strong negative correlation between large and small LDL might conceal the atherogenicity of large LDL.	Unclear. Large LDL is enriched in cholesterol, which may become cholesterol donors at some point.	Future studies should consider the inverse correlation between LDL subclasses to identify the true effect of large LDL-P.
LDL-S	LDL size	nm	LDL-S was found to be inversely associated with atherosclerosis (AS) and cardiovascular disease (CVD), reflecting similar but weaker information compared to LDL subclasses distribution.	The smaller LDL-S is, the more sdLDL particles may exist.	The relationship between LDL-S and atherosclerotic diseases was inconsistent or lost statistical significance after adjustments.
LDL-TG	Triglycerides in LDL	mg/dL	LDL-TG was proved to be positively associated with AS and subsequent CVD even after multivariable adjustments, but some studies failed to prove the association.	Unclear. LDL-TG may participate in the local inflammation after penetrating into arterial wall.	The number of studies focusing on LDL-TG using NMR is limited. Since TG is not riched in LDL, to which degree LDL-TG explains the risk and the exact mechanisms remain to be solved.
**Triglyceride-rich lipoprotein (TRL)**					
Small VLDL-P	Small VLDL particle number	nmol/L	Small VLDL-P was found to have a positive dose-response relationship with residual CVD risk, independent of LDL-C.	Small VLDL can diffuse into arterial wall without modification.	Whether small VLDL-P can serve as a therapeutic target needs more clinical and basic studies.
Large VLDL-P	Large VLDL particle number	nmol/L	Large VLDL-P was presented to be positively associated with AS and CVD in some studies, but the relationship was not supported in other studies.	Large VLDL may correlate with delayed chylomicron clearance, reflecting the postprandial lipemia.	The relationship between large VLDL and atherosclerotic diseases was discordant in different studies, and needs further researches.
RLP-C	Cholesterol in TRLs remnant	mg/dL	RLP-C, especially cholesterol in small VLDL, showed strong association with residual CVD risk.	Cholesterol in RLP, including small VLDL and IDL, exhibits increased retention time in artery wall.	The relationship between fasting and (or) postprandial RLP-C levels and CVD has been a research hotspot, and needs further confirmation in different populations.
VLDL-TG	Triglycerides in VLDL	mg/dL	VLDL-TG was not shown to explain CVD risk like VLDL-TC. But in insulin-resistant subjects, VLDL-TG usually increases, generating lipoprotein disturbances.	Insulin resistance induces increased TG-enriched VLDL, which leads to atherosclerotic lipoprotein profile.	Whether VLDL-TG accounts for CVD risk and how it exerts an adverse effect, remain to be investigated.
**High-density lipoprotein (HDL)**					
HDL-P	HDL particle number	nmol/L	HDL-P had a negative association with CVD, with a consistency across diverse ethnicities.	HDL is the only protective lipoprotein in circulation. Elevated HDL-P reflects efficient protection effects.	Whether HDL-P presents stable relationship with CVD across diverse ethnicities, ages, sex requires validation.
Smaller HDL-P	Smaller HDL particle number	nmol/L	Smaller HDL-P negatively correlated with CVD and improved the prediction efficiency.	Smaller HDL removes cholesterol from arterial wall, and has anti-inflammatory, antioxidant, antiapoptotic actions.	Several issues remain unclear: which HDL subclass better protects against CVD, the mechanisms of performing these
Large HDL-P	Large HDL particle number	nmol/L	Large HDL-P showed a inverse association with CVD risk.	Unclear. Larger HDL may also relate to the reverse cholesterol transport activity.	functionalities, how these actions are compromised in pathological states etc.
HDL-S	HDL size	nm	HDL-S inversely correlated with AS, but the relationship may be attenuated by other factors.	Unclear	HDL-S seems to be a weaker predictor than other HDL-related parameters.
HDL-ApoA1	apolipoproteinA-1 in HDL	mg/dL	ApoA1 strongly correlated with HDL-C, HDL-P, and offered extra risk information.	ApoA1 ensures HDL structural stability and cholesterol efflux.	Whether distribution of ApoA1 plays a part needs further research.
HDL-TG	Triglycerides in HDL	mg/dL	HDL-TG was reported to be directly associated with arteriosclerotic diseases.	Unclear	The mechanisms of HDL-TG exerting pathogenic effects require to be solved.

### LDL Particle Number, Subclasses, Particle Size, and Lipid Components

The LDL-C in standard lipid panel is calculated from the Friedewald equation which is easily influenced by hypertriglyceridemia (69), but qNMR provides quantitative data directly. In addition, the LDL-C only refers to cholesterol in LDL, while qNMR detects various LDL-related measures such as particle number, subclasses, particle size, and lipid components.

There has been agreement that LDL particle number (LDL-P) is strongly related to atherosclerotic diseases. In 2,888 individuals from the EPIC (European Prospective Investigation into Cancer and Nutrition)-Norfolk study, LDL-P correlated with coronary artery disease (CAD), even after adjusting for the Framingham risk score and LDL-C (70). Toth et al. (71) showed that patients undergoing LDL-P measurement were more likely to receive intensive lipid-lowering therapy and had lower CVD risk than those in the LDL-C cohort. In fact, LDL-C and LDL-P may be discordant in some cases, exerting an additional risk (72–74). In the MESA (Multi-Ethnic Study of Atherosclerosis) study, Otvos et al. (72) identified 5,598 participants with discordant LDL-P and LDL-C. The carotid intima-media thickness (cIMT) was highest in subjects with raised LDL-P but normal LDL-C. Among 27,533 initially healthy women that were followed up for 17.2 years in another study (74), a similar result was observed, namely, coronary risk was underestimated for women with such discordance. Therefore, as described in the National Lipid Association (NLA) consensus statement (75), LDL-P is a “reasonable measure” in the estimation and management of CVD risk and may be a potential intervention target.

In regard to LDL subclasses, it is well-established that small dense LDLs (sdLDLs) are more atherogenic (76–79). In fact, the necessity of monitoring the sdLDL concentration has been stressed by many guidelines, such as the 2016 Chinese guidelines (80) and 2019 ESC/EAS guidelines (81). However, some researchers regard the large LDL subclass to be as important as sdLDL. Mora et al. (82) found that failure to adjust the strong inverse association between sdLDL and large LDL might conceal the atherogenic effect of large LDL. Likewise, Otvos et al. (83) demonstrated that both LDL subclasses were significantly related to coronary events once their correlation was taken into account. Moreover, it is worth noting that LDL particle size (LDL-S) in nanometers (nm) reflects similar but weaker information compared to the LDL subclass distribution. Specifically, although LDL-S was shown to be negatively associated with atherosclerotic diseases in some studies (84, 85), the relationship was not sustained in other studies or lost its statistical significance when adjusting for other lipid parameters (82, 86).

With regard to lipid components in LDL, most of the current studies targeting triglycerides in LDL (LDL-TG) use other methods, such as the enzyme-linked immunosorbent assay (ELISA) kits and automated homogeneous methods (87, 88). For example, Saeed et al. (89) examined plasma LDL-TG levels with automated homogeneity and discovered that LDL-TG independently correlated with the incidence of CVD events. In 4,381 patients with established CAD, high LDL-TG was associated with worse cardiovascular outcomes, suggesting its utility among secondary prevention populations (90). Likewise, Tzoulaki et al. (91) assessed metabolic profiling among 3,867 MESA participants based on qNMR metabolomics. The results indicated that TGs in total and large (density 1.019–1.031 kg/L) LDL were related to cIMT and CVD events (*P* < 0.005) when adjusting for other risk factors. Nevertheless, Albers et al. (92) failed to prove the correlation between LDL-TG and CVD events in the AIM-HIGH (Atherothrombosis Intervention in Metabolic Syndrome with Low HDL/High Triglycerides and Impact on Global Health Outcomes) trial. Thus, more studies using qNMR to precisely quantify LDL-TG levels are needed to explore its atherogenicity.

Although the mechanisms underlying the atherosclerotic process that are secondary to dyslipidemia remain largely unclear, the adverse effects of LDL have been partially identified. Circulating LDLs penetrate endothelial cells and enter the intimal space, especially in the context of hypercholesterolemia (93, 94). After being exposed to oxidative injury by free radical species, modified LDL can then be engulfed by monocyte-derived macrophages through receptor-mediated endocytosis and phagocytosis (95, 96). Macrophages subsequently turn into foam cells engorged with large amounts of cholesteryl esters, which give rise to the initiation of vascular lesions and the formation of fatty streaks (97). Regarding lipid components in LDL, it is interesting to note that TG can be catabolized by macrophages, which is different from cholesterol (98). LDL-TG may not directly lead to plaque formation, but may still potentiate atherogenesis. Elevated LDL-TG correlated with inflammatory markers such as C-reactive protein, white blood cell count, amyloid-A, fibrinogen, and interleukin-6 (89, 99) and was negatively associated with adiponectin levels, which have anti-inflammatory and cardioprotective effects (100). Therefore, chronic low-grade inflammation may be the link between LDL-TG and atherosclerosis, and more possible mechanisms remain under active investigation.

### VLDL Subclasses, and Lipid Components in VLDL and IDL

Increasing attentions have been focused on TG-rich lipoproteins, which consist of VLDL, IDL in the fasting state, and plus chylomicron (CM) in the postprandial state (101–103). Since CM particles may generate NMR spectral interference, qNMR detections usually avoid nonfasting serum or plasma, we thereby discuss the fasting TRLs, namely, VLDL and IDL in this part.

The first thing to mention is the concentrations of VLDL subclasses. Early in 1998, Freedman et al. (104) found that large (diameter 60–100 nm) VLDL was positively associated with CAD independent of age and standard lipid measurements. Four years later, Mackey et al. (105) reported that the level of large (diameter 60–200 nm) VLDL was associated with higher coronary calcification (CAC) after multivariable adjustments. However, studies performed in the JUPITER (Justification for the Use of Statins in Prevention) trial seemed to endorse the smaller VLDL subclasses. In 11,984 participants with a baseline LDL-C lower than 3.36 mmol/l (130 mg/dl), each standard deviation (SD) increase in the concentration of small (diameter 29–42 nm) VLDLs resulted in a 68% increase in residual CVD risk (106). However, statin-induced changes in large (diameter > 60 nm) or medium (diameter 42–60 nm) VLDL particles caused no risk reduction (107). In addition to the two opposite views, some studies considered all VLDL subclasses to have adverse effects. Mora et al. (108) found that total VLDL and its subclasses, as well as IDL, all independently predicted the incidence of CVD events in 27,673 women that were followed up for over 11 years. In 4,662 individuals from the CKB (China Kadoorie Biobank) cohort, the adjusted odds ratio (OR) for myocardial infarction ranged from 1.18 (1.07–1.29) for extremely large VLDL to 1.30 (1.19–1.44) for small VLDL, including IDL in the interval (109).

The second point that needs to be discussed is lipid components in VLDL and IDL. In recent years, cholesterol in TRL remnants (RLP-C), mainly small VLDL-C, has been proven to play a crucial role in residual CVD risk. Lawler et al. (107) observed a dose-dependent relationship between a decrease in VLDL-C and the residual risk reduction. In addition to VLDL-C, VLDL-TG is also an index of great concern but is under debate. In 25,480 Copenhagen subjects, Balling et al. (110) discovered that VLDL-TG did not account for MI risk, whereas VLDL-C explained 50% of the risk among ApoB-containing lipoproteins. However, for the same CVD endpoint, Holmes et al. (109) found that TG in all VLDL subclasses (diameter from 31.3 to >75 nm) had strong positive correlations with MI. Similarly, Tzoulaki et al. (91) demonstrated that nearly all VLDL-TG correlated with cIMT and TG in total and that the largest VLDL was related to CVD after multivariable adjustments (*P* < 0.005). Hence, VLDL-TG is very likely to take part in the atherogenic process and should be considered seriously in future studies.

Despite belonging to ApoB-containing lipoproteins, large VLDL, owing to the size limitation of transcytotic vesicles, cannot traverse the endothelium and exert atherogenic effects like LDL (111). However, some studies considered elevated large VLDL might reflect postprandial dyslipidemia, which is characterized by delayed chylomicron clearance and prolonged atherogenic lipoprotein retention (105). In addition, basic studies suggested that small RLP particles, which contain 5–20 times more cholesterol than LDL, had a greater affinity for subendothelial components. The TRL remnants could be taken up by macrophages directly without modification, thus promoting rapid cholesterol accumulation in lesional macrophages (22, 112). TRL remnants are also proposed to exacerbate the atherosclerotic process by inducing the secretion of TNF-α and IL-1β (113), activating the coagulation cascade (114), impairing endothelium-dependent vasodilation (115), and increasing oxidative stress (116). Except for its own pathogenicity, elevated VLDL under insulin resistance can also initiate lipoprotein destabilization (117). Specifically, excess VLDL particles undergo deficient TG lipolysis and poor hepatic uptake, which results in increased RLP particles. Afterwards, VLDL particles and remnants promote TG transfer to LDL and HDL by CETP. The latter two particles consequently become TG-enriched and can be further hydrolyzed into more atherogenic sdLDL and small TG-depleted, TC-depleted HDL (118). The whole pathological disturbances alter the lipoprotein profile towards an atherogenic form. In summary, TRL subclasses and their lipid composition promote endothelial dysfunction, but further research is still needed to identify the underlying mechanisms.

### HDL Particle Number, Subclasses, Particle Size, and Compositional Components

Since HDL is not simply a carrier of cholesterol but a complex particle with physiological heterogeneity, the structural, and compositional parameters measured by qNMR such as particle number, particle size, subclasses, and compositional components tend to better reflect the cardioprotective effects of HDL.

In the MESA cohort, HDL particle number (HDL-P) was protective against cIMT and CHD after adjusting for HDL-C, LDL-C, LDL-P, TG, and other confounders (119). In the Dallas Heart Study, when HDL-C was no longer related to CVD in multivariable analyses, HDL-P maintained a strong correlation (120). With no interaction with black race, HDL-P had consistency across diverse ethnicities (121). Moreover, among four HDL-related biomarkers in the JUPITER trial, HDL-P was consistently the strongest predictor of CVD at baseline or on statins (122). These studies all support that HDL-P might be a promising protective biomarker in atherosclerotic diseases.

The HDL subclass that best reflects the antiatherogenic features of HDL has been laden with controversy. Some studies describe large HDL as an effective form. A nested case-control study in the CKB cohort discovered that all HDL subclasses, except for small (diameter < 8.7 nm) HDL, were negatively associated with MI (109). In the Chicago Healthy Aging Study, large (diameter 8.8–13 nm) and medium (diameter 8.2–8.8 nm) HDL particle levels positively correlated with HDL cholesterol efflux capacity (CEC), which characterizes the key function of effluxing cellular cholesterol (123). Nevertheless, conflicting results have been reported. Ditah et al. (124) suggested that medium (diameter 8.2–9.4 nm) and small (diameter 7.2–8.2 nm) HDL were associated with CAC even after adjusting for HDL-C, while large (diameter 9.4–14 nm) HDL fell short of statistical significance. Silbernagel et al. (125) presented an inverse correlation of small (diameter 7.0–8.5 nm) HDL with CVD mortality, which could further improve the performance of the prediction model. Kim et al. (126) found that small and medium HDL-P was strongly associated with cardioprotective paraoxonase 1 (PON1) activity and that PON1 is a glycoprotein enzyme that prevents oxidation of LDL. These studies fuel the speculation that small HDL subclasses act as more accurate risk indicators.

HDL particle size (HDL-S) is usually inversely correlated with AS, but the negative relationship might be attenuated or abolished by other factors. In the Women's Health Study, the adjusted hazard ratio (HR) for HDL-S was 0.65 (0.51–0.81) (108). However, in the EPIC study, the association between HDL-S and CAD was diminished after adjusting for ApoB and TG levels (127). In the JUPITER trial, HDL-S underwent slight drug-induced changes and showed no association with CVD in fully adjusted models (128). As a result, in the long run, HDL-S seems to be a weaker predictor than the HDL subclass distribution.

In addition to HDL quantity, HDL quality has aroused growing attention. HDL functions are closely related to its compositional components. ApoA1 is the major protein on HDL that ensures structural stability and stimulates cholesterol efflux from cells to HDL (32). In the *post-hoc* analysis of the IDEAL (Incremental Decrease in End Points through Aggressive Lipid Lowering) trial and EPIC-Norfolk study, when HDL-C lost its inverse relationship with major coronary events after serial adjustments, ApoA1 exhibited a stable negative association in most models (129). Moreover, it also suggested that high levels of very large HDL particles not accompanied by high levels of ApoA1 were associated with increased but not decreased CVD risk (129). Therefore, ApoA1 should be taken into account more in risk assessment. Regarding lipid components in HDL, most cohort studies indicated that the cholesterol in small HDL subclasses primarily drove the inverse association with CVD events (130, 131). However, there is also a study that supports the idea that the cholesterol in large HDL inversely correlates with MI (109). Furthermore, despite the protective properties of HDL on the whole, HDL-TG has been reported to be positively correlated with arteriosclerotic diseases in many populations, such as the CKB cohort (109), MESA cohort (91) and T2DM or MS patients (132). Thus, it may be a potential pathogenic biomarker of CVD risk.

HDL particles demonstrate multiple antiatherogenic biological functions. The most widely known is reverse cholesterol transport (RCT) (133). Small HDL particles remove cholesterol from the arterial wall, primarily from the lipid-overloaded macrophages, and gradually expand into larger spherical HDL. These large HDL particles transfer their cholesterol esters to hepatocytes by scavenger receptor-B1 (SR-B1) or to ApoB-containing lipoproteins using CETP, regenerating a smaller HDL that repeats the process. Additional atheroprotective properties of HDL, particularly the small and medium HDL subclasses, include angiectatic, anti-inflammatory, antioxidant, and antiapoptotic actions. Specifically, HDL can stimulate the production of nitric oxide (NO) and prostacyclin to exert vasodilatory effects (134). HDL also suppresses the chronic inflammatory response by decreasing the generation of adhesion molecules, platelet-activating factor and Von Willebrand factor (135). By reducing the production of reactive oxygen species and contrasting the oxidation of LDL through paraoxonase or platelet activating factor acetyl hydrolase (136, 137), HDL is able to decrease intracellular oxidative stress. Furthermore, HDL particles exert cytoprotective actions, protecting both macrophages and endothelial cells from apoptosis (138). Taken together, small HDL particles seem to mainly represent the protective capacity of HDL, but more evidence from experimental studies *in vitro* and *in vivo* is needed.

### qNMR-Based Molecular Metabolites

The risk assessments of atherosclerotic diseases are traditionally based on lipoproteins and lipids. However, several qNMR platforms also enable the simultaneous detection of low-molecular-weight metabolites, offering multimetabolic signatures. In this review, we briefly introduce some typical qNMR-based molecular biomarkers.

Glycoprotein acetyls (GlycA) is a novel NMR biomarker of systemic inflammation that reflects the enzymatic glycosylation state of the main acute-phase reactants. Compared with hsCRP, GlycA was reported to have lower analytic imprecision and intraindividual variability (139). In the MESA cohort, GlycA was associated not only with subclinical atherosclerosis (140–142) but also with poorer CVD health independent of hsCRP, d-dimer, IL-6, and fibrinogen (143). However, it is not clear whether GlycA is a determinant or just an indicator of atherosclerotic progression.

The amino acid profile is also included in the qNMR metabolome, even though its overall picture may not be as clear as the lipoprotein profile. In the Taizhou Imaging Study, two branched-chain amino acids (leucine and isoleucine) were positively correlated with arterial stiffness (144). Additionally, phenylalanine showed an inverse association with CVD (91). In another multiqueue study, a high phenylalanine level was consistently associated with CVD death but was only associated with CVD events at a young age, except in elderly individuals over 60 years (145).

Lactate was inversely associated with cIMT in the Taizhou Imaging Study (144). But in another multi-racial study, lactate showed positive associations with CVD events (91). With respect to fatty acids, higher levels of monounsaturated fatty acid (MUFA) were positively correlated with CVD risk while higher levels of omega-6 fatty acids and docosahexaenoic acid (DHA) negatively related to CVD risk (145).

## Significance of qNMR-Based Metabolites in Atherosclerotic Diseases

A statement by the American Heart Association addressed the potential impact of metabolomics on CVD health and disease (146). Indeed, studies looking for novel biomarkers using qNMR metabolomics, as described in the previous section, usually have three major, interlinked objectives, which will be discussed below.

### Improving Risk Assessment

Current CVD risk assessments, such as the Framingham Risk Score (FRS) (147) and the China-PAR tool (148), rely on traditional risk factors (TRFs). However, the first CVD event often originates in people classified as being at intermediate or low risk; hence, it is hoped that new qNMR-based metabolite biomarkers can complement or outperform the existing risk estimations.

The weighted metabolite score derived from 13 replicated signals was independently associated with CHD incidence. When adding age and sex to the score, the predictive performance paralleled that of TRFs (C-index 0.81 and 0.82) (149). The model using the calculated “VLDL extra-hepatic lipolysis indicator” and “VLDL hepatic turnover indicator” combined with LDL-C and HDL-C had better CVD risk prediction performance than that with only TRFs (AUROC 0.812 vs. 0.795) (150). In addition, in a meta-analysis across three cohorts, the prediction score incorporating phenylalanine, MUFAs, omega-6 fatty acids and DHA improved risk classification, particularly for people in the intermediate (5–10%) risk range (145).

Thus, qNMR metabolites not only offer additional risk information beyond traditional lipids but also have the clinical potential to reclassify the CVD risk stratification. In other words, qNMR metabolomics can be taken as an extension to routine analysis and, to a certain degree, may supersede lipid panels in the future.

### Unraveling Disease Etiology

qNMR metabolomics is expected to shed light on the “black box” mechanisms of AS and CVD. Biological pathways underlying the biomarkers can be characterized by searching metabolic pathway databases such as the Kyoto Encyclopedia of Genes and Genomes (KEGG; *https://www.kegg.jp*) and Human Metabolome Database (HMDB; *https://www.hmdb.ca*) (151). Clinical studies and basic research studies have shown that during AS progression, metabolic perturbations lie in lipid and fatty acid metabolism, BCAA and aromatic acid metabolism, the TCA cycle, glycolysis, urea metabolism, oxidative stress, and inflammatory and insulin pathways (152–154). Mapping the molecular interaction gene network can further depict interconnections between disturbed pathways to gain a more precise understanding (91).

Exposure to various environmental factors may bring about metabolic changes contributing to atherogenesis. A meta-analysis of 26,065 individuals from eight cohorts reported that age-specific metabolic profiles differed by sex, and the menopausal transition in females induced a proatherogenic lipoprotein profile along with amino acid changes (155). Additionally, the association between HDL-C and CVD events was attenuated in patients with chronic CAD or chronic kidney disease, probably owing to long-term inflammation, oxidative stress, and abnormal hormone secretion (38). In addition, a polymorphism of the *APOA5* gene, which encodes a key regulator of TG levels, could modify lipoprotein distributions toward a more atherogenic pattern. This pattern was significantly associated with increased cIMT, especially in overweight or centrally obese patients, thus reflecting a “double hit” from gene-environment interactions (118).

### Guiding Therapeutic Strategy

The qNMR-based metabolic profile is capable of monitoring treatment efficacy. For example, among subjects treated with gemfibrozil, a fibric acid derivative, neither TG nor HDL-C predicted CHD events, but LDL-P and HDL-P could serve as independent predictive indexes (83). Therefore, the therapeutic benefit not reflected by conventional biochemical testing may be uncovered by qNMR metabolomics.

The profiling of metabolic changes also contributes to personalized intervention or optimal evaluation. Six metabolic patterns were identified in the early stage of AS (156). Among them, phenotype A held the highest risk (RR = 2.6); phenotype B presented less lipoprotein variation than A (RR = 2.4). Phenotype C held a relatively optimal profile, but the risk (RR = 1.8) was not accordingly desirable. For phenotypes D, E, and F, the relative risks were statistically similar. Thus, various metabolic phenotypes may involve varying risk levels. Proper classification of AS patients can help direct individualized treatment. Moreover, in the JUPITER trial that recruited subjects with low LDL-C, a small (diameter 29–42 nm) VLDL-P was responsible for residual CVD risk (106), and HDL-P was the strongest inverse predictor for CVD among several HDL-related indexes (122). Hence, qNMR metabolic profiles allow for the identification of potential targets for intervention and prognostic indicators for evaluation.

## Challenges and Future Directions of qNMR Metabolomics Studies in Atherosclerotic Diseases

Although great progress has been made in regard to techniques, including automatic sample injection, spectral feature extraction, and efficient analytical tools, studies using qNMR metabolomics in AS and subsequent CVD still face a series of issues. In this section, we discuss several challenging points.

### Identifying Key Metabolite Biomarkers

It is required to determine the “golden value” biomarkers for clinical applications. An eligible biomaker should fulfill some prerequisites, including valid and precise measurement, additional value beyond existing tests, and clinical benefit to subjects (157, 158).

NMR metabolomics offers metabolic profiles in reproducible manners. The relative standard deviations (RSD) of metabolites derived from qNMR spectroscopy are much smaller (i.e., values are within less fluctuation range) than those detected by mass spectrometry (57). In this regard, qNMR metabolomics endows its biomarkers with some practicability.

Despite a plethora of biomarkers for AS and CVD that have been identified in epidemiological studies, very few successfully find their way into clinical use. Various biomarker panels comprise different lipoproteins and/or molecular metabolites, which bring great difficulties in comparison and validation. Another point that needs to be noted is the application scope. Although AS is a systemic disease, risk factors across differing arterial beds and atherosclerotic endpoints do not necessarily overlap (159, 160). Thus, owing to intergroup variability and disease complexity, few biomarker candidates have been translated into clinical practice, and further research is still required.

In addition, the cost-benefit needs to be considered. NMR metabolomics detects a suite of metabolites. Making a diagnosis based on so many metabolites would not be convenient or economical in clinical practice. Encouragingly, the simplified LipoProfile panel (including LDL particle number, NMR determined HDL-C, and TG) was approved by the U.S. Food and Drug Administration in 2008 (161) and was covered by some health insurance companies in America. However, the clinical utilization rates and practical benefits remain unclear. If the clinical benefits are very small or deficient, the additional costs may not be justified.

### Various Analytical Platforms

The lack of standardized and uniform settings has become one of the biggest barriers to putting biomarker candidates into practice. A variety of analytical methods have been developed to separate lipoprotein subfractions according to their size or density. However, the measurement units differ between qNMR platforms, which makes it difficult to compare or combine results from different studies.

However, on the upside, a series of consortia or programs have been established to overcome the heterogeneity. The Consortium of Metabolomics Studies (COMETS) was developed in 2014 (162). As the largest consortium worldwide, it builds on 47 prospective studies that include over 136,000 participants from Asia, Europe, North America, and South America as of April 2018. Furthermore, the Atheroflux consortium consists of two earlier EU consortia, AtheroRemo and RiskyCAD (157). The intention of these alliances is to combine multiple data and to discern more reliable targets for clinical treatment and scientific research. It can be anticipated that with more communication among researchers, uniform criteria or comparative methods across various platforms may be available in the future.

### Necessity of Collaboration With Other Methodologies

Despite the many advantages of qNMR, especially the unique ability to quantify lipoproteins, its sensitivity is relatively poor in contrast to that of mass spectrometry. It is difficult to identify molecules with concentrations much lower than the detection limit; thus, qNMR is currently less preferred than MS in lipidomics. Moreover, the high sample volume requirement makes it difficult to perform assays on specimens at trace levels. The overlapping resonances in spectral regions may correspond to multiple metabolites, posing a great challenge to metabolite annotation (45). By comparison, MS has a greater sensitivity and can identify complex mixtures, despite its suboptimal quality control and high cost of absolute quantification. Therefore, NMR and MS have become complementary technologies in the field of metabolomics.

It should be noted that qNMR metabolomics provides only a “snapshot” of metabolic profiles. To learn about the potential causal pathways and upstream changes, cooperation with genomics, transcriptomics, proteomics, and microbiomics is required to identify serial biomarkers (38, 158). Additionally, metabolomics studies may only function as hypothesis-generating to provide an initial framework for further research. Whether the identified biomarkers are pathogenic and through which signaling pathways the biomarkers play their role remain to be solved. Hence, to elucidate definitive pathological mechanisms underlying the atherogenic process, more scientific investigations need to be performed.

## Conclusion

In the postgenomic era, qNMR metabolomics has been widely applied in atherosclerotic diseases, which allows for the rapid, accurate and high-throughput measurements of circulating lipoproteins, lipids, and some molecular metabolites. In this review, we summarized the recent qNMR metabolomics studies associated with AS and CVD, with a particular emphasis on lipoprotein biomarkers referring to particle number, particle size, and compositional components. Since various qNMR platforms have discordant categorization criteria, along with intraindividual variability, interlab heterogeneity, and confounding factor interference, few biomarker candidates have been ultimately accepted in routine clinical practice. In conclusion, more extensive exploration in large population-based cohorts and validation of clinical applications should be pursued in the future. To successfully translate the “golden value” metabolite biomarkers from bench to bedside, we still have a long way to go.

## Author Contributions

SM drafted the manuscript. MX and XG revised, researched, and discussed the article. All authors approved it for publication.

## Conflict of Interest

The authors declare that the research was conducted in the absence of any commercial or financial relationships that could be construed as a potential conflict of interest.

## Publisher's Note

All claims expressed in this article are solely those of the authors and do not necessarily represent those of their affiliated organizations, or those of the publisher, the editors and the reviewers. Any product that may be evaluated in this article, or claim that may be made by its manufacturer, is not guaranteed or endorsed by the publisher.
