# Supplementary material for: Biomarker Discovery in Atherosclerotic Diseases Using Quantitative Nuclear Magnetic Resonance Metabolomics
Source: Front Cardiovasc Med. 2021 Jul 28;8:681444. doi: 10.3389/fcvm.2021.681444 (PMC8356911; doi:10.3389/fcvm.2021.681444)
Supplement: Supplementary file 1 [file Table_1.pdf]

Table S1

Epidemiological Studies of Subclinical Atherosclerosis Using Quantitative NMR Metabolomics

Year	Study Design	Subject	Disease	Main Findings	NMR Platform	Reference
2021	cross-sectional analysis	112 women (28 T1DM and pre-eclampsia; 28 T1DM; 28 pre-eclampsia; 28 controls)	cIMT and carotid plaque	Medium VLDL-P, HDL-C/HDL-TG correlated with carotid plaque in pre-eclampsia. HDL-C/HDL-P was associated with maximum cIMT in T1DM.	Liposcale test	Amor et al.(1)
2020	cross-sectional analysis	489 individuals in Taizhou Longitudinal Study	PWV and cIMT	Leucine and isoleucine, acetoacetate, two lipids (CH2C=C, CH2COO) positively correlated with PWV. Lactate was inversely associated with cIMT.	NMR spectrometer (Bruker Biospin, Germany)	Jiang et al.(2)
2019	multiqueue, cohort study	3,867 participants in MESA; 3,569 participants in Rotterdam and LOLIPOP	CAC, cIMT and incident CVD (MI, stroke)	TC, ApoB, LDL-ApoB, histidine correlated with both CAC and cIMT. LDL1-TG, VLDL6-PL, TG, ApoB, acetaminophen glucuronide were associated with CVD.	NMR spectrometer (Bruker Biospin, Germany)	Tzoulaki et al.(3)
2019	cross-sectional analysis	935 men (589 HIV-infected; 346 HIV-uninfected)	subclinical coronary plaque	Higher GlycA was associated with a higher prevalence of CAC>0 and coronary stenosis ≥50%.	LipoScience, Inc. (now LabCorp, Raleigh, NC)	Tibuakuu et al.(4)
2019	multiqueue, cross-sectional study	1,178 children and 1,316 parents in CheckPoint; 4,249 children and 4,171 mothers in ALSPAC	cIMT and PWV	Glucose, some HDL-C derived measures, glutamine, histidine, tyrosine correlated with PWV. However in children, no metabolites were consistently associated with cIMT and PWV.	Nightingale NMR metabolomics platform (Helsinki, Finland)	Juonala et al.(5)
2019	cross-sectional analysis	502 patients with T2DM or MS	carotid plaque	HDL-TG was directly associated with worsening metabolism and carotid plaque in high CVD risk patients.	Liposcale test	Girona et al.(6)
2019	cohort study	6,466 participants in MESA	ABI, carotid plaque and PAD	Higher GlycA was associated with prevalent low ABI and carotid plaque, and could predicted incident PAD.	LipoScience, Inc. (now LabCorp, Raleigh, NC)	Fashanu et al.(7)
2018	cross-sectional analysis	177 children with familial hypercholesterolaemia	cIMT	Children with familial hypercholesterolaemia had increased total, and small LDL particles, which directly correlated with cIMT.	Liposcale test	Rodríguez-Borjabad et al.(8)

2017	cross-sectional analysis	656 subjects (214 lean; 228 obese; and 214 T2DM)	a self-developed vascular score	NMR lipid measures were better indicators for vascular outcomes than traditional lipid measures.	LipoScience, Inc. (now LabCorp, Raleigh, NC)	Urbina et al.(9)
2017	cross-sectional analysis	402 participants in CHAS	lipid-rich necrotic core plaque	HDL efflux capacity and HDL-P were inversely associated with lipid-rich necrotic core plaque in unadjusted models. HDL-S was not related to the plaque.	LipoScience, Inc. (now LabCorp, Raleigh, NC)	Mutharasan et al.(10)
2016	cross-sectional analysis	214 obese youth (96 prediabetes; 118 normoglycemic) in MESA	cIMT and PWV	Youth with prediabetes exhibited higher small LDL-P, small HDL-P; lower intermediate and large HDL-P, which independently correlated with arterial thickness and stiffness.	LipoScience, Inc. (now LabCorp, Raleigh, NC)	Shah et al.(11)
2016	multiqueue, cross-sectional study	6,512 participants in MESA and 126 men in CLEAR	cIMT	Small+medium HDL-P inversely correlated with common and internal cIMT even after adjusting for HDL-C. The cardioprotective effect of smaller HDL particles might relate to paraoxonase 1 activity.	LipoScience, Inc. (now LabCorp, Raleigh, NC)	Kim et al.(12)
2016	cross-sectional analysis	504 Jerusalem residents	CAC	Total, medium+small HDL-P were strongly associated with CAC rather than HDL-C, large HDL-P, HDL-S.	LipoScience, Inc. (now LabCorp, Raleigh, NC)	Ditah et al.(13)

Abbreviations: ABI, ankle-brachial index; ApoB, apolipoprotein B; BMI, body mass index; BP, blood pressure; CAC, coronary artery calcium; CHD, coronary heart disease; cIMT, carotid intima-media thickness; CVD, cardiovascular diseases; GlycA, Glycoprotein acetyls; HDL, high-density lipoprotein; HDL-C, cholesterol in HDL; HDL-P, HDL particle number; HDL-S, HDL particle size; HDL-TG, triglyceride in HDL; HIV, human immunodeficiency virus; LDL, low-density lipoprotein; LDL-ApoB, ApoB in LDL; LDL-P, LDL particle number; LDL-TG, triglyceride in LDL; MI, myocardial infarction; MS, metabolic syndrome; NMR, nuclear magnetic resonance; PAD, Peripheral artery disease; PWV, pulse wave velocity; T1DM, Type 1 diabetes mellitus; T2DM, Type 2 diabetes mellitus; TC, total cholesterol; TG, triglyceride; VLDL, very-low-density lipoprotein; VLDL-P, VLDL particle number; VLDL-PL, phospholipid in VLDL.

Table S2

Epidemiological Studies of Cardiovascular Disease Using Quantitative NMR Metabolomics

Year	Study Design	Subject	Disease	Main Findings	NMR Platform	Reference
2020	meta-analysis	38,797 participants from seven prospective cohorts	incident stroke	Medium HDL-C, large and medium LDL-TG, histidine, pyruvate, GlycA were associated with stroke after multivariable adjustments.	LipoScience (Raleigh, NC); Nightingale platform (Helsinki, Finland)	Vojinovic et al.(14)
2020	cohort study	214 men with stable CAD followed up for 12.5 years	CVD mortality	Total, small HDL-P showed the strongest inverse associations with all-cause and CVD mortality.	NMR (Bruker Biospin, Germany)	Duparc et al.(15)
2020	cohort study	109,751 individuals followed up for 11 years	myocardial infarction	VLDL-C explained 50% of the MI risk from ApoB-containing lipoproteins, but VLDL-TG did not account for the risk.	Nightingale NMR metabolomics platform (Helsinki, Finland)	Balling et al.(16)
2019	experimental study	58 T2DM patients completing a 6-month CR diet intervention	lipid-related CVD risk	After intervention, participants had increased LDL-S, HDL-C, decreased small LDL-P, TG and TG/HDL.	LipoScience, Inc. (now LabCorp, Raleigh, NC)	Mason et al.(17)
2018	cohort study	1162 adult followed up for 12.4 years	incident CVD (CHD, stroke)	Higher medium and small LDL-P were associated with increased CVD risk, especially for CHD.	Liposcale test	Pichler et al.(18)
2018	nested case-control study	4,662 individuals (912 MI; 1,146 IS; 1,138 IH; 1,466 control) in CKB	incident MI, IS, ICH	Lipoprotein patterns were similar in MI and IS, but not with ICH. HDL-TG was positively associated with MI. Glycoprotein acetyls and several non–lipid-related metabolites were associated with all 3 diseases.	NMR metabolomics method developed by Ala-Korpela	Holmes et al.(19)
2018	cross-sectional analysis	6,479 participants in MESA	CVD health	GlycA was inversely associated with CVD health assessed by Life's Simple 7 scores.	LipoScience, Inc. (now LabCorp, Raleigh, NC)	Benson et al.(20)
2017	cohort study	2,290 participants followed up over a median of 10 years	CVD mortality	Total HDL-P, mainly small HDL-P, inversely related to CVD mortality. Adding either of the two measurements rather than HDL-C to multivariate prediction models improved performance.	NMR at Numares AG (previously known as LipoFIT GmbH)	Silbernagel et al.(21)

2017	cohort study	27,533 initially healthy women followed up over a median of 20.4 years	coronary events	Discordance between non-HDL-C and ApoB or non-HDL-C and LDL-P occurred in many healthy women. In such conditions, ApoB or LDL-P might better predict CHD risk.	LipoScience, Inc. (now LabCorp, Raleigh, NC)	Lawler et al.(22)
2017	experimental study	9,423 participants in JUPITER trial	incident CVD end point	Small VLDL decreases showed a dose-response relationship with residual risk reductions, independent of LDL-C changes.	LipoScience, Inc. (now LabCorp, Raleigh, NC)	Lawler et al.(23)
2017	multiqueue, cohort study	11,984 participants in JUPITER trial; 4721 individuals in CATHGEN	incident CVD end point	On-statin levels of small VLDL positively related to residual CVD risk, which might derive from VLDL-C but not total TG or larger VLDL. Similar pattern was observed in the CATHGEN cohort.	LipoScience, Inc. (now LabCorp, Raleigh, NC)	Lawler et al.(24)
2017	nested case-control study	638 participants (314 CVD cases; 314 controls) in JUPITER trial	incident CVD end point	HDL-P was consistently the strongest inverse predictor of CVD events at baseline and on-statin.	LipoScience, Inc. (now LabCorp, Raleigh, NC)	Khera et al.(25)
2016	nested case-control study	6,417 participants (1,596 MS; 838 DM; 3,983 control) in MESA	incident CHD and CVD	Subjects with MS or DM had much more LDL or HDL particle-cholesterol discordance. The LDL discordance and higher LDL-P in MS could predict CHD and CVD, as was the higher LDL-C and lower HDL-P in DM.	LipoScience, Inc. (now LabCorp, Raleigh, NC)	Tehrani et al.(26)

Abbreviations: ApoA1, apolipoprotein A-I; ApoB, apolipoprotein B; CAC, coronary artery calcium; CHD, coronary heart disease; cIMT, carotid intima-media thickness; CVD, cardiovascular diseases; GlycA, Glycoprotein acetyls; HDL, high-density lipoprotein; HDL-C, cholesterol in HDL; HDL-P, HDL particle number; HDL-TG, triglyceride in HDL; ICH, intracerebral hemorrhage; IDL, intermediate density lipoprotein; IS, ischemic stroke; LDL, low-density lipoprotein; LDL-C, cholesterol in LDL; LDL-P, LDL particle number; LDL-S, LDL particle size; LDL-TG, triglyceride in LDL; MI, myocardial infarction; MS, metabolic syndrome; NMR, nuclear magnetic resonance; non-HDL-C, non-HDL cholesterol; SD, standard deviation; T2DM, Type 2 diabetes mellitus; TC, total cholesterol; TG, triglyceride; VLDL, very-low-density lipoprotein; VLDL-C, cholesterol in VLDL; VLDL-P, VLDL particle number; VLDL-TG, triglyceride in VLDL.

References

1. Amor AJ, Vinagre I, Valverde M, Urquizu X, Meler E, López E, et al. Nuclear magnetic resonance lipoproteins are associated with carotid atherosclerosis in type 1 diabetes and pre-eclampsia. *Diabetes Metab Res Rev* (2021) **37**:e3362. doi:10.1002/dmrr.3362
2. Jiang Y, Zhang K, Zhu Z, Cui M, An Y, Wang Y, et al. Associations between serum metabolites and subclinical atherosclerosis in a Chinese population: the Taizhou Imaging Study. *Aging* (2020) **12**:15302–15313. doi:10.18632/aging.103456
3. Tzoulaki I, Castagné R, Boulangé CL, Karaman I, Chekmeneva E, Evangelou E, et al. Serum metabolic signatures of coronary and carotid atherosclerosis and subsequent cardiovascular disease. *Eur Heart J* (2019) **40**:2883–2896. doi:10.1093/eurheartj/ehz235
4. Tibuakuu M, Fashanu OE, Zhao D, Otvos JD, Brown TT, Haberlen SA, et al. GlycA, a novel inflammatory marker, is associated with subclinical coronary disease. *AIDS Lond Engl* (2019) **33**:547–557. doi:10.1097/QAD.0000000000002079
5. Juonala M, Ellul S, Lawlor DA, Santos Ferreira DL, Carlin JB, Cheung M, et al. A Cross-Cohort Study Examining the Associations of Metabolomic Profile and Subclinical Atherosclerosis in Children and Their Parents: The Child Health CheckPoint Study and Avon Longitudinal Study of Parents and Children. *J Am Heart Assoc* (2019) **8**:e011852. doi:10.1161/JAHA.118.011852
6. Girona J, Amigó N, Ibarretxe D, Plana N, Rodríguez-Borjabad C, Heras M, et al. HDL Triglycerides: A New Marker of Metabolic and Cardiovascular Risk. *Int J Mol Sci* (2019) **20**: doi:10.3390/ijms20133151
7. Fashanu OE, Oyenuga AO, Zhao D, Tibuakuu M, Mora S, Otvos JD, et al. GlycA, a Novel Inflammatory Marker and Its Association With Peripheral Arterial Disease and Carotid Plaque: The Multi-Ethnic Study of Atherosclerosis. *Angiology* (2019) **70**:737–746. doi:10.1177/0003319719845185
8. Rodríguez-Borjabad C, Ibarretxe D, Girona J, Ferré R, Feliu A, Amigó N, et al. Lipoprotein profile assessed by 2D-1H-NMR and subclinical atherosclerosis in children with familial hypercholesterolaemia. *Atherosclerosis* (2018) **270**:117–122. doi:10.1016/j.atherosclerosis.2018.01.040
9. Urbina E, McCoy C, Gao Z, Khoury P, Shah A, Dolan L, et al. Lipoprotein Particle Number & Size Predict Vascular Structure & Function Better than Traditional Lipids in Adolescents & Young Adults. *J Clin Lipidol* (2017) **11**:1023–1031. doi:10.1016/j.jacl.2017.05.011
10. Mutharasan RK, Thaxton CS, Berry J, Daviglus ML, Yuan C, Sun J, et al. HDL efflux capacity, HDL particle size, and high-risk carotid atherosclerosis in a cohort of asymptomatic older adults: the Chicago Healthy Aging Study. *J Lipid Res* (2017) **58**:600–606. doi:10.1194/jlr.P069039
11. Shah AS, Davidson WS, Gao Z, Dolan LM, Kimball TR, Urbina EM. Superiority of lipoprotein particle number to detect associations with arterial thickness and stiffness in obese youth with and without prediabetes. *J Clin Lipidol* (2016) **10**:610–618. doi:10.1016/j.jacl.2016.01.007
12. Kim DS, Li YK, Bell GA, Burt AA, Vaisar T, Hutchins PM, et al. Concentration of Smaller High-Density Lipoprotein Particle (HDL-P) Is Inversely Correlated With Carotid Intima Media Thickening After Confounder Adjustment: The Multi Ethnic Study of Atherosclerosis (MESA). *J Am Heart Assoc Cardiovasc Cerebrovasc Dis* (2016) **5**: doi:10.1161/JAHA.115.002977

13. Ditah C, Otvos J, Nassar H, Shaham D, Sinnreich R, Kark JD. Small and medium sized HDL particles are protectively associated with coronary calcification in a cross-sectional population-based sample. *Atherosclerosis* (2016) **251**:124–131. doi:10.1016/j.atherosclerosis.2016.06.010
14. Vojinovic D, Kalaoja M, Trompet S, Fischer K, Shipley MJ, Li S, et al. Association of circulating metabolites in plasma or serum and risk of stroke: Meta-analysis from seven prospective cohorts. *Neurology* (2020) doi:10.1212/WNL.0000000000011236
15. Duparc T, Ruidavets J-B, Genoux A, Ingueneau C, Najib S, Ferrières J, et al. Serum level of HDL particles are independently associated with long-term prognosis in patients with coronary artery disease: The GENES study. *Sci Rep* (2020) **10**:8138. doi:10.1038/s41598-020-65100-2
16. Balling M, Afzal S, Varbo A, Langsted A, Davey Smith G, Nordestgaard BG. VLDL Cholesterol Accounts for One-Half of the Risk of Myocardial Infarction Associated With apoB-Containing Lipoproteins. *J Am Coll Cardiol* (2020) **76**:2725–2735. doi:10.1016/j.jacc.2020.09.610
17. Mason AE, Saslow LR, Moran PJ, Kim S, Abousleiman H, Richler R, et al. Lipid findings from the Diabetes Education to Lower Insulin, Sugars, and Hunger (DELISH) Study. *Nutr Metab* (2019) **16**:58. doi:10.1186/s12986-019-0383-2
18. Pichler G, Amigo N, Tellez-Plaza M, Pardo-Cea MA, Dominguez-Lucas A, Marrachelli VG, et al. LDL particle size and composition and incident cardiovascular disease in a South-European population: The Hortega-Liposcale Follow-up Study. *Int J Cardiol* (2018) **264**:172–178. doi:10.1016/j.ijcard.2018.03.128
19. Holmes MV, Millwood IY, Kartsonaki C, Hill MR, Bennett DA, Boxall R, et al. Lipids, Lipoproteins, and Metabolites and Risk of Myocardial Infarction and Stroke. *J Am Coll Cardiol* (2018) **71**:620–632. doi:10.1016/j.jacc.2017.12.006
20. Benson E-MA, Tibuakuu M, Zhao D, Akinkuolie AO, Otvos JD, Duprez DA, et al. Associations of ideal cardiovascular health with GlycA, a novel inflammatory marker: The Multi-Ethnic Study of Atherosclerosis. *Clin Cardiol* (2018) **41**:1439–1445. doi:10.1002/clc.23069
21. Silbernagel G, Pagel P, Pfahlert V, Genser B, Scharnagl H, Kleber ME, et al. High-Density Lipoprotein Subclasses, Coronary Artery Disease, and Cardiovascular Mortality. *Clin Chem* (2017) **63**:1886–1896. doi:10.1373/clinchem.2017.275636
22. Lawler PR, Akinkuolie AO, Ridker PM, Sniderman AD, Buring JE, Glynn RJ, et al. Discordance between Circulating Atherogenic Cholesterol Mass and Lipoprotein Particle Concentration in Relation to Future Coronary Events in Women. *Clin Chem* (2017) **63**:870–879. doi:10.1373/clinchem.2016.264515
23. Lawler PR, Akinkuolie AO, Harada P, Glynn RJ, Chasman DI, Ridker PM, et al. Residual Risk of Atherosclerotic Cardiovascular Events in Relation to Reductions in Very-Low-Density Lipoproteins. *J Am Heart Assoc Cardiovasc Cerebrovasc Dis* (2017) **6**: doi:10.1161/JAHA.117.007402
24. Lawler PR, Akinkuolie AO, Chu AY, Shah SH, Kraus WE, Craig D, et al. Atherogenic Lipoprotein Determinants of Cardiovascular Disease and Residual Risk Among Individuals With Low Low-Density Lipoprotein Cholesterol. *J Am Heart Assoc Cardiovasc Cerebrovasc Dis* (2017) **6**: doi:10.1161/JAHA.117.005549
25. Khera AV, Demler OV, Adelman SJ, Collins HL, Glynn RJ, Ridker PM, et al. Cholesterol Efflux Capacity, High-Density Lipoprotein Particle Number, and Incident Cardiovascular Events: An Analysis From the JUPITER Trial (Justification for the Use of Statins in Prevention: An

Intervention Trial Evaluating Rosuvastatin). *Circulation* (2017) **135**:2494–2504. doi:10.1161/CIRCULATIONAHA.116.025678

26. Tehrani DM, Zhao Y, Blaha MJ, Mora S, Mackey RH, Michos ED, et al. Discordance of Low-Density Lipoprotein and High-Density Lipoprotein Cholesterol Particle Versus Cholesterol Concentration for the Prediction of Cardiovascular Disease in Patients With Metabolic Syndrome and Diabetes Mellitus (from the Multi-Ethnic Study of Atherosclerosis [MESA]). *Am J Cardiol* (2016) **117**:1921–1927. doi:10.1016/j.amjcard.2016.03.040
